# Humeral Avulsion of the Lateral Collateral Ligament of the Elbow Concomitant with the Medial Epicondyle Fracture of a Child with General Joint Laxity

**DOI:** 10.1155/2019/1965343

**Published:** 2019-04-04

**Authors:** Toru Morimoto, Masashi Izumi, Hiroaki Ueba, Masahiko Ikeuchi

**Affiliations:** Department of Orthopaedic Surgery, Kochi University, Kochi Medical School, Kohasu, Oko-cho, Nankoku 783-8505, Japan

## Abstract

Medial epicondyle fracture is a common elbow injury for children, and it was reported that 30-50% of this fracture was associated with elbow dislocation. However, dysfunction of the lateral collateral ligament (LCL) complex accompanied with the medial epicondyle fracture has rarely been reported. In this report, a 13-year-old girl who had a humeral avulsion of the LCL concomitant with a displaced medial epicondyle fracture was presented. Since her general joint laxity had been recognized from past medical history and the humeral avulsion of the LCL was clearly confirmed by ultrasonography, simultaneous surgical repair for the LCL avulsion and medial epicondyle fracture was conducted and satisfactory outcome was achieved. Although general joint laxity does not tend to receive attention in trauma as much as chronic conditions, it should be borne in mind to prevent overlooking important soft tissue damage coexisting with apparent fractures.

## 1. Introduction

Medial epicondyle fracture is the most common avulsion injury of the elbow and accounts for 11-20% of all pediatric elbow fractures [1–3]. It occurs most frequently between the ages of 9 and 14 and is four times more common in boys [4]. Reviews of previous literatures described that medial epicondyle fractures were associated with elbow dislocation in 30-50% of children [5]; however, there were very few reports highlighting possible concurrent elbow injury such as coronoid fracture, radial head fracture, and lateral collateral ligament avulsion. In this report, we present a rare case of humeral avulsion of the lateral collateral ligament (LCL) concomitant with the medial epicondyle fracture of a young girl, which was presumably associated with her general joint laxity.

## 2. Case Report

A 13-year-old right hand-dominant girl had fallen on her hand and injured the right elbow during her way to school and visited our clinic soon after. Her chief complaint was a severe pain of the right elbow that was apparently swollen. Active and passive motion of the elbow was not possible due to pain, but no neurovascular deficits were observed in her left arm. She had a history of dwarfism and had been undergoing growth hormone replacement therapy since she was 7 years old at our hospital. She also had a history of developmental dysplasia of the left hip and a habitual patellar dislocation of the right knee. For the patellar dislocation, she underwent reconstructive surgery of the medial patellofemoral ligament when she was 12 years old, and at that time, her general joint laxity was diagnosed with the Beighton score of 6 points.

**Figure 1:**
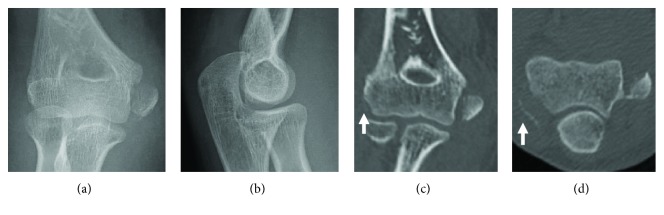
Preoperative X-ray (a, b) and CT (c, d) of the right elbow. (a) AP view. (b) Lateral view. (c) Coronal plane. (d) Axial plane. Thin bony fragment of the lateral epicondyle (arrow) was observed in (c) and (d).

In plain X-ray and CT scan of the elbow, apparent displaced medial epicondyle fracture (Watson-Jones classification type 2) together with an avulsed thin fragment of the lateral epicondyle was detected; however, no proximal radial fracture or a coronoid fracture was observed (Figure 1). She could not undergo MRI because of her panic disorder and claustrophobia.

**Figure 2:**
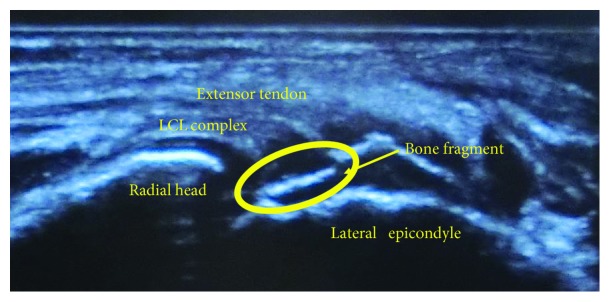
Preoperative ultrasonography of the right elbow. Humeral avulsion of the LCL was clearly confirmed.

**Figure 3:**
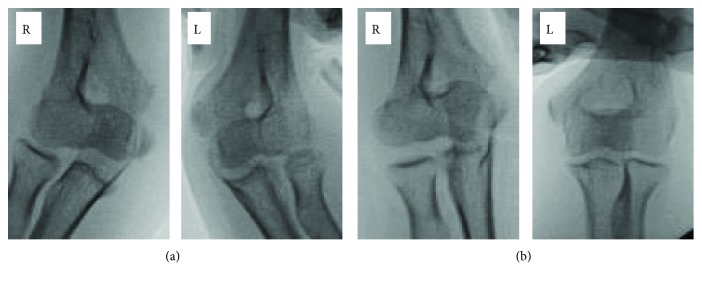
Preoperative stress X-ray of the bilateral elbow evaluated under anesthesia. (a) Valgus. (b) Varus. (R: right elbow, L: left elbow.) The side-to-side differences of the joint space widening were significantly worse in the right elbow.

The next day after the injury, her surgery was performed under general anesthesia. Prior to skin incision, ultrasonography and stress X-rays of the elbow were evaluated. Proximal avulsion of LCL from the epicondyle was clearly seen in the ultrasonography (Figure 2). Valgus and varus stress tests were performed bilaterally with her elbows at a 20-degree flexed position. The side-to-side differences of the joint space widening were 8 mm in the valgus stress and 3 mm in the varus stress, which were significantly worse in the right elbow (Figure 3).

**Figure 4:**
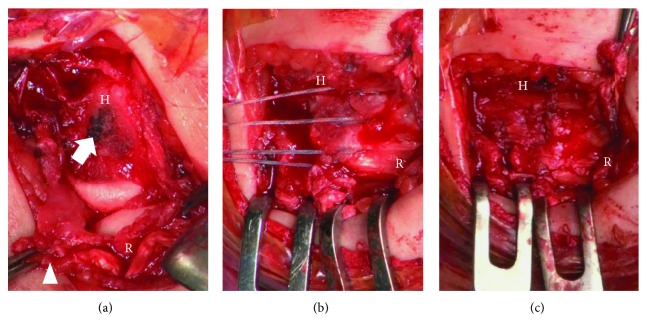
Intraoperative photos. Upper: cranial, lower: caudal. H: humerus; R: radius. (a) Humeral avulsion of the LCL (arrow: foot print, arrow head: LCL complex). (b) Suture preparation. (c) After repair.

For the medial epicondyle fracture, conventional open reduction and internal fixation was performed using tension band wiring technique. Then, the lateral epicondyle was exposed and there was a complete avulsion of the LCL complex but the conjoined extensor tendon was not involved. After refreshing the stump of the ligament and footprint, 3 suture anchors (*φ*1.4 mm JuggerKnot®, Zimmer Biomet, Warsaw, IN, US) were inserted into the footprint and LCL complex was sutured by double row fixation technique (Figure 4).

**Figure 5:**
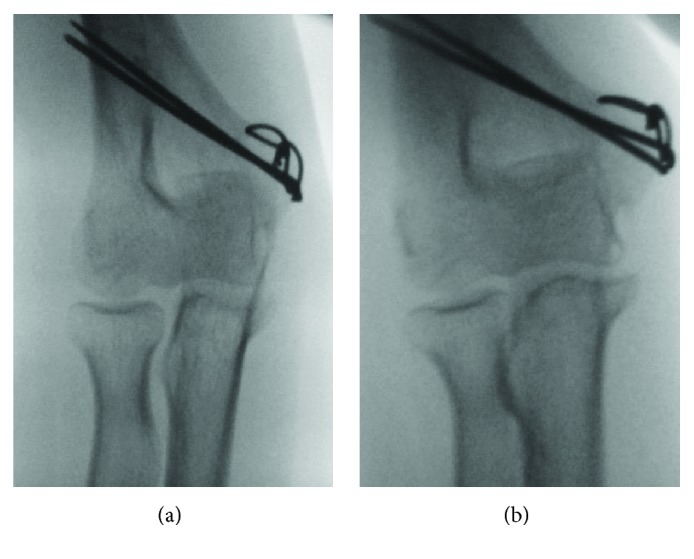
Postoperative stress X-ray of the right elbow evaluated under anesthesia (4 months after the primary surgery). There was no difference of the instability.

Postoperatively, her elbow was immobilized with a plastic cast for 3 weeks and then was allowed active and passive motion with a soft brace. She was able to return to her preinjury daily activities 2 months after the surgery. The wires were removed 4 months after the primary surgery. At that time, the medial epicondyle fracture was united, and side-to-side differences of the joint space widening were 2 mm in the valgus stress and 1 mm in the varus stress, which had significantly improved compared with the previous findings (Figure 5). Her follow-up was completed 7 months after the primary surgery, with no pain, no limitation of ROM, and no functional deficits due to the elbow injury.

## 3. Discussion

**Figure 6:**
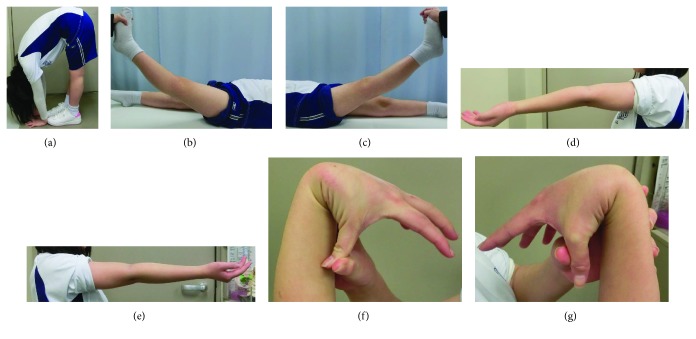
Her general joint laxity. (a) Spine. (b) Left knee. (c) Right knee. (d) Left elbow. (e) Right elbow. (f) Left wrist. (g) Right wrist. All photos were taken at the point of final follow-up so that the right knee (c) and the right elbow (e) did not show hyperextension due to surgical intervention.

A rare child case of elbow injury was successfully treated with simultaneous surgical repair for LCL avulsion and medial epicondyle fracture. In this case, general joint laxity was certainly a key of diagnosis and treatment (Figure 6). Basically, the general joint laxity is essential information for diagnosing and determining treatment plan of instability-associated chronic joint disorders such as recurrent dislocation of the shoulder and patella [6–8], so experienced orthopedic surgeons usually keep in mind when they see a juvenile patient with joint instability. However, it does not tend to receive attention in trauma as much as chronic conditions, especially in cases with apparent and typical fracture, and is often overlooked by important soft tissue injury. In our case, her general joint laxity had been recognized from the past medical history, which provided more suitable assessments particularly regarding LCL injury and subsequent optimal treatment.

As mentioned, half of pediatric medial epicondyle fractures is associated with elbow dislocation [5]. According to cadaveric biomechanical studies, rotatory forces accompanied with valgus or varus stress are essential to the elbow dislocation mechanism, which typically results in medial and lateral collateral ligament avulsion at the humeral insertion [9]. In our case, it was unclear whether dislocation had occurred or not and whether it had naturally reduced before visiting the hospital because typical dislocation-associated fractures such as coronoid fracture and proximal radius fracture were not observed. However, humeral avulsion of the LCL concomitant with medial epicondyle fracture strongly suggested that abnormal varus and valgus forces were applied at the time of injury, which was possibly exaggerated by her original joint laxity. Though most of medial epicondyle fractures will have some ligament damages and not every case needs to fix the LCL complex, it would be beneficial to mind the possibility of severe LCL injury especially in cases with general joint laxity.

It should be discussed that treatment option of LCL avulsion, i.e., nonsurgical treatment with elbow immobilization following the repair of medial epicondyle fracture, might be a reasonable management for this injury. However, in our case, simultaneous surgical repair of the LCL was recommended because of the following reasons: (1) she had originally lax ligaments around her joints, (2) humeral LCL avulsion was strongly suspected from the preoperative CT and clearly confirmed by the ultrasonography, and (3) significant side-to-side difference of lateral instability was documented from the stress X-rays evaluated under anesthesia. It has been well known that posterolateral rotatory instability of the elbow after dislocation is one of the most significant causes of terrible dysfunction in daily and sports activities [9, 10], and a primary repair of the LCL complex could possibly reduce risk for developing residual instability [11]. Though the follow-up period was relatively short in this report, our case showed satisfactory clinical and radiological results after the simultaneous surgical repair.

MRI should be a gold standard to assess ligament injury; however, ultrasonography is a good alternative that can provide both static and dynamic evaluations specifically for a site of ligament avulsion. In our case, it was hard to carry out a MRI because of her panic disorder and claustrophobia; nevertheless, avulsed LCL complex combined with unstable thin bony fragment was clearly confirmed by the ultrasonography before the skin incision, which encouraged us for determining the treatment plan. In addition, displaced medial epicondyle fracture did not cause any disturbance for the evaluation of the LCL complex. Of course, not every medial epicondyle fracture case needs ultrasonography, but it seems to be the most suitable evaluation to prevent overlooking important soft tissue damage coexisting with apparent elbow fractures in children.
